# Baseline characteristics of the North American prodromal Synucleinopathy cohort

**DOI:** 10.1002/acn3.51738

**Published:** 2023-02-08

**Authors:** Jonathan E. Elliott, Miranda M. Lim, Allison T. Keil, Ronald B. Postuma, Amelie Pelletier, Jean‐François Gagnon, Erik K. St. Louis, Leah K. Forsberg, Julie A. Fields, Daniel E. Huddleston, Donald L. Bliwise, Alon Y. Avidan, Michael J. Howell, Carlos H. Schenck, Jennifer McLeland, Susan R. Criswell, Aleksandar Videnovic, Emmanuel H. During, Mitchell G. Miglis, David R. Shprecher, Joyce K. Lee‐Iannotti, Bradley F. Boeve, Yo‐El S. Ju, the North American Prodromal Synucleinopathy (NAPS) Consortium, Yo‐El S. Ju, Bradley F. Boeve, Alon Y. Avidan, Donald L. Bliwise, Parichita Choudhury, Susan R. Criswell, Emmanuel H. During, Jonathan E. Elliott, Julie A. Fields, Leah K. Forsberg, Jean‐François Gagnon, Michael J. Howell, Daniel E. Huddleston, Joyce K. Lee‐Iannotti, Miranda M. Lim, Mitchell G. Miglis, Ronald B. Postuma, David R. Shprecher, Erik K. St. Louis, Aleksandar Videnovic, Jennifer McLeland, Sommer Amudson‐Huffmaster, Nellie Brushaber, Jae Woo Chung, Joshua De Kam, Adrian Ekelmans, Marissa Keane, Allison T. Keil, Ruth Kraft, Brittany R. Ligman, Colum MacKinnon, Daeva Miner‐Rose, Samantha Murphy, Amelie Pelletier, Katherine L. M. Powers, Matthew Stauder, Adreanne Rivera, Sarahmay Sanchez, Rebekah Summers, Luke Tiegan, Paul Timm, Kelsey A. Tucker, Peter Tran, Douglas Galasko, Emmanuel Mignot, Carlos Schenck

**Affiliations:** ^1^ VA Portland Health Care System Research Service Portland Oregon USA; ^2^ Oregon Health & Science University Neurology, Portland Oregon USA; ^3^ Behavioral Neuroscience Oregon Health & Science University Portland Oregon USA; ^4^ Department of Pulmonary and Critical Care Medicine Oregon Health & Science University Portland Oregon USA; ^5^ Oregon Institute of Occupational Health Sciences Oregon Health & Science University Portland Oregon USA; ^6^ Neurology VA Portland Health Care System Portland Oregon USA; ^7^ Mental Illness Research Education and Clinical Center VA Portland Health Care System Portland Oregon USA; ^8^ National Center for Rehabilitative Auditory Research VA Portland Health Care System Portland Oregon USA; ^9^ Montreal Neurological Institute McGill University Montreal Québec Canada; ^10^ Psychology Université du Québec à Montréal Montreal Québec Canada; ^11^ Hôpital du Sacré‐Coeur de Montréal Center for Advanced Research in Sleep Medicine Montreal Québec Canada; ^12^ Mayo Clinic, Neurology and Medicine Rochester Minnesota USA; ^13^ Neurology Emory University Atlanta Georgia USA; ^14^ Neurology, Sleep Disorders Center University of California Los Angeles Los Angeles California USA; ^15^ Neurology University of Minnesota Medical Center Minneapolis Minnesota USA; ^16^ Hennepin County Medical Center, Minnesota Regional Sleep Disorders Center Minneapolis Minnesota USA; ^17^ Washington University School of Medicine St. Louis Missouri USA; ^18^ Movement Disorders Unit, Division of Sleep Medicine Massachusetts General Hospital Boston Massachusetts USA; ^19^ Harvard Medical School Neurological Clinical Research Institute Boston Massachusetts USA; ^20^ Psychiatry and Behavioral Sciences Stanford University Redwood City California USA; ^21^ Neurology & Neurological Sciences Stanford University Palo Alto California USA; ^22^ Neurology Banner Sun Health Research Institute Phoenix Arizona USA

## Abstract

**Objective:**

Rapid eye movement (REM) sleep behavior disorder (RBD) is widely considered a prodromal synucleinopathy, as most with RBD develop overt synucleinopathy within ~10 years. Accordingly, RBD offers an opportunity to test potential treatments at the earliest stages of synucleinopathy. The North American Prodromal Synucleinopathy (NAPS) Consortium has created a multisite RBD participant, primarily clinic‐based cohort to better understand characteristics at diagnosis, and in future work, identify predictors of phenoconversion, develop synucleinopathy biomarkers, and enable early stage clinical trial enrollment.

**Methods:**

Participants ≥18 years of age with overnight polysomnogram‐confirmed RBD without Parkinson's disease, dementia, multiple system atrophy, or narcolepsy were enrolled from nine sites across North America (8/2018 to 4/2021). Data collection included family/personal history of RBD and standardized assessments of cognitive, motor, sensory, and autonomic function.

**Results:**

Outcomes are primarily reported based on sex (361 total: *n* = 295 male, *n* = 66 female), and secondarily based on history of antidepressant use (*n* = 200 with, *n* = 154 without; with correction for sex differences) and based on extent of synucleinopathy burden (*n* = 56 defined as isolated RBD, *n* = 305 defined as RBD+ [i.e., exhibiting ≥1 abnormality]). Overall, these participants commonly demonstrated abnormalities in global cognition (MoCA; 38%), motor function (alternate tap test; 48%), sensory (BSIT; 57%), autonomic function (orthostatic hypotension, 38.8%), and anxiety/depression (BAI and PHQ‐9; 39.3% and 31%, respectively).

**Interpretation:**

These RBD participants, assessed with extensive history, demographic, cognitive, motor, sensory, and autonomic function demonstrated a lack of sex differences and high frequency of concomitant neurological abnormalities. These participants will be valuable for future longitudinal study and neuroprotective clinical trials.

## Introduction

The North American Prodromal Synucleinopathy (NAPS) Consortium for Rapid Eye Movement (REM) sleep behavior disorder (RBD) was established to facilitate neuroprotective clinical trials for neurodegenerative diseases characterized by synuclein pathology. Such “synucleinopathies,” including Parkinson's disease (PD), dementia with Lewy bodies (DLB), and multiple system atrophy (MSA), affect ~2‐million people in the US, have a staggering economic footprint,^1^ and lack treatments to prevent inevitable severe disability and death. To date, all neuroprotective therapies (defined by disease‐modifying treatments) for synucleinopathy patients have failed,^2^, ^3^ with one possible explanation being that the pathological changes present at time of clinical diagnosis were already too advanced and no longer modifiable, or otherwise did not modify the underlying clinical syndrome. Treatments may have greater chance of efficacy during the prodromal stage (i.e., synuclein pathology prior to overt symptoms). Currently, there are no established/widely used biomarkers for detecting prodromal synucleinopathies.

RBD is a disorder characterized by a lack of muscle atonia during REM sleep and dream enactment behaviors,^4^, ^5^ and is strongly associated with synucleinopathies.^6^, ^7^, ^8^, ^9^ In addition to a high proportion of individuals with overt synucleinopathies having RBD, even among individuals with “idiopathic” RBD, 40%–70% phenoconvert/develop an overt synucleinopathy within 5–10 years.^10^, ^11^, ^12^ (henceforth “RBD” will be used for RBD occurring in the absence of neurological diseases. Additionally, since the term “isolated RBD” has been used interchangeably with “idiopathic RBD” in the literature, we note that the NAPS Consortium defines “isolated RBD” differently, i.e., without accompanying signs/symptoms of neurodegeneration, as detailed in the Methods section). Single‐center studies have demonstrated that many individuals with RBD exhibit incipient abnormalities in motor function, cognition, autonomic function, color vision, olfaction, and brain imaging, similar to but milder than in overt synucleinopathies.^13^, ^14^ Abnormal synuclein aggregates in the enteric nervous system, submandibular glands, and skin have been reported in RBD.^15^, ^16^, ^17^, ^18^ Taken together, RBD should be considered a prodromal stage of an evolving synucleinopathy in most individuals ≥50 years old, which could suggest RBD may offer an opportunity to test potential treatments at early stages of synucleinopathy, when disease progression may be more susceptible to modification.

Building upon existing RBD research groups (e.g., the International RBD Study Group),^19^ the NAPS Consortium (https://www.naps‐rbd.org) comprises a coordinated effort across nine sites in North America to establish an RBD cohort prospectively assessed by standardized assessments and biomarker collection. Herein we describe the baseline characteristics of the NAPS Consortium, a primarily but not exclusively clinic‐based cohort, which at present, reflecting a single cross‐sectional timepoint (prospective, longitudinal follow‐up on this cohort are ongoing). Data are presented describing the whole cohort, with special attention placed on sex, whether prodromal synucleinopathy features are present, antidepressant use, and family history, given that each of these traits have proved meaningful in past studies.

## Materials and methods

### Overview

A complete methodological description of the NAPS Consortium protocol is published in a separate overview paper (Ju et al. Pending). Briefly, participants >18 years of age with polysomnogram‐confirmed RBD by ICSD‐3 criteria^20^ who did not have a diagnosis of PD,^21^ dementia of any type,^22^ MSA,^23^ or narcolepsy^24^ were enrolled from nine sites across North America. From August 2018 to April 2021, total *n* = 361 participants were enrolled at Washington University School of Medicine (*n* = 26), Mayo Clinic Rochester (*n* = 50), University of Minnesota (*n* = 30), Center of Advanced Research in Sleep Medicine at the *Hôpital du Sacré‐Coeur de Montréal* (*n* = 94), Harvard/Massachusetts General Hospital (*n* = 26), Emory University (*n* = 32), University of California Los Angeles (*n* = 32), Stanford University (*n* = 20), and the VA Portland Health Care System (*n* = 51). Participants from a tenth site, at Banner Sun Health, are not included in this analysis. Referrals were made primarily from clinics offered on a consecutive basis and supplemented by community referrals through the www.naps‐rbd.org website. There were no *a priori* demographic for goals recruitment, and information regarding those who chose not to participate was not retained. This study was performed according to the *Declaration of Helsinki* and approved by the Institutional Review Boards at each enrollment site. All participants provided written informed consent prior to participation.

Data collection procedures and practices were rigorously standardized across sites, which included structured interviews and questionnaires on health history, structured neurological, and physical examinations, and an objective test battery of cognitive, motor, autonomic, and sensory functions. If available, a co‐participant (a spouse, family member, or friend who knows the participant well) provided information via structured interviews and written questionnaires. Variables presented in this analysis are detailed below; according to the NAPS1 REDCap database export dated May 26, 2022 prior to complete import into the NAPS2 RAVE database. Biofluid assay data, polysomnograms, data from participants enrolling after April 2021, and data from longitudinal study visits are not presented in this analysis.

Based on the clinical evaluation, questionnaires, exam, and objective tests, the clinician at each site (a board‐certified neurologist) completed a standardized, structured assessment to render clinical diagnoses. Determinations were supported by a quarterly, panel‐based adjudication process with three or more NAPS clinicians. Participants were categorized as “RBD+” indicating one or more abnormalities (symptoms, signs, or test results attributable to a neurodegenerative cause) in any of the cognitive, motor, autonomic, or sensory domains (*n* = 305); or as “isolated RBD” indicating no abnormalities were detected during the clinical evaluation and test battery (*n* = 56). Participants with diagnosis of any overt neurodegenerative disease (*n* = 6; rare, since a previously known diagnosis would have been exclusionary) are excluded from this analysis.

### RBD and sleep measures

A NAPS‐specific structured interview queried for RBD symptoms, frequency, severity, treatments, and possible temporal relationship with any antidepressant or other medications. Diagnoses of sleep apnea (obstructive or central), restless legs syndrome, and periodic limb movement were determined during the clinician's structured interview. The severity and frequency of RBD symptoms was assessed by the RBD Severity Scale (RBDSS),^25^ completed by the participant as well as bedpartner (RBDSS‐BP), if available. Other sleep‐related measures included the Epworth Sleepiness Scale (ESS) to measure daytime sleepiness^26^ and the Scales for Outcomes in PD‐Sleep (SCOPA‐Sleep) which queries nighttime sleep quality and daytime sleepiness.^27^

### Other health history and questionnaires

Demographic information and health history (including comprehensive family history) were obtained via structured interview and forms, including those from the Uniform Data Set version 3 (UDS3), from the National Alzheimer Coordinating Center (https://naccdata.org/data‐collection/forms‐documentation/uds‐3) and custom NAPS‐specific forms. Questionnaires assessing neuropsychiatric function included the Beck Anxiety Inventory (BAI), Patient Health Questionnaire‐9 (PHQ‐9) for depression, the Post‐Traumatic Stress Disorder (PTSD) checklist (PCL‐5),^28^ and an informant‐completed Neuropsychiatric Inventory‐Questionnaire (NPI‐Q).^29^ Autonomic function was assessed using the Scales for Outcomes in PD‐Autonomic Dysfunction (SCOPA‐AUT).^30^

### Neurological test battery

Participants underwent a broad neurological test battery including objective tests of cognitive, motor, autonomic, and sensory (color vision and smell) function. Cognitive assessments included the psychometric battery from the UDS3 standard and LBD modules^31^: Montreal Cognitive Assessment (MoCA),^32^ the Craft Story 21 (immediate and delayed), the Benson Complex Figure Copy (immediate and delayed), Number Span Test Forward and Backward, Trail Making Test parts A and B, categorical and phenomic fluency (animals, vegetables, words beginning with F and L), Multilingual Naming Test (MINT), the Speeded Attention Task,^33^ and the Noise Pareidolia Task.^34^, ^35^ Raw scores were adjusted for age, sex, and years of education, and Z‐scored. Scores ≥ 1.5 standard deviations below the mean were considered abnormal, and two abnormal tests in a domain (memory, attention/executive, visuospatial, or language) were required for the domain to be considered abnormal.

Motor function was assessed via the Timed Up and Go (TUG),^36^ Purdue Pegboard,^37^ and Alternate Tap tests. Collectively, these assessments evaluated participants' gross motor function, ability to sit/stand/walk, fine motor control and coordination of the limbs and digits, and overall reaction/movement speed. Additionally, the Movement Disorders Society Unified PD Rating Scale (MDS‐UPDRS) was administered, and part 3 of this assessment includes a clinician‐quantified rating of gross and fine motor function.^38^

Autonomic function was measured with orthostatic blood pressure, in which blood pressure was measured after lying supine for 5 min, then 3 min after standing. Impaired orthostatic tolerance was defined as ≥20 mm Hg decrease in systolic blood pressure and/or a ≥ 10 mm Hg decrease in diastolic blood pressure. Severe orthostatic intolerance was defined as ≥30 mm Hg decrease in systolic blood pressure and/or a ≥ 15 mm Hg decrease in diastolic blood pressure.^39^, ^40^ Additionally, subjective autonomic function was assessed using the Scales for Outcomes in PD—Autonomic Dysfunction (SCOPA‐AUT).^30^

The Brief Smell Identification Test (B‐SIT; Version A, Sensonics Inc. NJ, USA)^41^ assessed overall olfaction and scent discrimination, with higher scores indicating greater olfactory function. Sex‐ and age‐adjusted cutoffs were used to define abnormal results, however, in general scores ≤8 indicate impaired olfaction.^42^ The Farnsworth Munsell 100 Color Hue test (FM‐100)^43^ assessed participants color discrimination ability, with higher scores indicating worse color vision. Age‐adjusted cutoff were used for FM‐100 scores, however, in general scores >100 suggest poor color discrimination.^44^ Participants self‐reported any color blindness or subjective smell impairment.

### Statistical analyses

Statistical analyses were performed with SPSS and GraphPad Prism v9, with alpha at 0.05. Data are presented as mean, standard deviation, number, and percentage of the whole. Data for the whole group (Tables 1, 2, 3, 4) are presented as descriptive statistics. Special emphasis was placed on the description of these data considering sex (Tables 1, 2, and 5, 6) and is similarly presented with comparisons between groups analyzed with either unpaired two‐tailed Student's *t* test, Mann–Whitney *U* test, or *χ* ^2^ test, as appropriate. Group differences between sex within RBD and sleep‐related outcomes (Table 2) were corrected for antidepressant usage defined as past or current self‐reported antidepressant use using a generalized linear model univariate analysis. These data are also shown based on two sub‐analyses, (1) with participants separated based on their self‐reported history of antidepressant usage (i.e., participants with any past or current use vs. participants who have never used antidepressants; Table 3), and (2) with participants separated based on their clinician determined degree of synucleinopathy burden and evidence of neurodegeneration (i.e., isolated RBD vs. RBD+; Table 4), with comparisons between groups analyzed with either unpaired two‐tailed Student's *t* test, Mann–Whitney *U* test, or *χ* ^2^ test, as appropriate. Throughout the presentation of results, *p*‐values should be interpreted with the understanding that corrections for multiple testing were not done and that potential issues surrounding selection and confounding were not robustly modeled.

**Table 1:** Demographic characteristics.

	Whole Cohort	Male	Female
	*n* = 361	*n* = 295	*n* = 66
Age, *years*	64.9 ± 10.1	65.2 ± 10.1	63.5 ± 9.7
Age, range (median; IQR)	28–85 (67; 11)	28–85 (68; 12)	30–81 (64; 13)
Handedness, *right*	305 (85%)	248 (84%)	58 (88%)
Ethnicity and race			
Ethnicity, *Hispanic/Latinx*	12 (3%)	9 (3%)	3 (5%)
Race, *White*	326 (90%)	266 (90%)	60 (91%)
Race, *Black or African American*	8 (2%)	7 (2%)	1 (1%)
Race, *other*	27 (8%)	22 (8%)	5 (8%)
Education			
Education, *≤12 years*	56 (16%)	48 (16%)	9 (14%)
Education, *13–14 years*	51 (14%)	41 (14%)	11 (16%)
Education, *15–18 years*	185 (52%)	147 (50%)	38 (58%)
Education, *≥19 years*	65 (18%)	59 (20%)	8 (12%)
Marital status			
Marital status, *married/domestic partner*	306 (85%)	236 (88%)	42 (70%)*
Marital status, *divorced/separated*	33 (9%)	21 (7%)	12 (18%)*
Marital status, *widowed*	9 (2%)	5 (2%)	4 (6%)*
Marital status, *never married/annulled*	13 (4%)	9 (3%)	4 (6%)
Living situation			
Living with spouse/partner	311 (86%)	265 (90%)	48 (73%)
Living alone	39 (11%)	24 (8%)	14 (21%)
Living with friend/roommate	11 (3%)	6 (2%)	4 (6%)
Living independently	344 (95%)	283 (96%)	62 (94%)
Living with some assistance	17 (5%)	12 (4%)	4 (6%)
Living in single/multi‐family residence	356 (99%)	289 (98%)	66 (100%)
Living in community/group living	4 (1%)	6 (2%)	0 (0%)

Data are presented as mean ± standard deviation, or raw frequency count with percent of the total number of available responses in parentheses.

* *p* < 0.05 female versus male.

**Table 2:** RBD and other sleep characteristics.

	Whole Cohort	Male	Female
	*n* = 361	*n* = 295	*n* = 66
RBD history			
Earliest age of onset, *years*	51.1 ± 16.3	51.6 ± 18.1	48.9 ± 21.0
Movement/talking	352 (98%)	288 (98%)	64 (97%)
Movement/talking w/ dreams, *always*	203 (56%)	164 (56%)	39 (59%)
RBD behavior without medication, *daily*	124 (34%)	110 (37%)	14 (21%)*
RBD behavior with medication, *daily*	80 (22%)	73 (25%)	7 (11%)*
RBD behavior: injured self, *ever*	202 (56%)	173 (59%)	29 (44%)
RBD behavior: injured self, *past 6 m*o	85 (24%)	76 (26%)	9 (14%)
RBD behavior: injured bed partner, *ever*	143 (40%)	132 (45%)	11 (17%)*
RBD behavior: injured bed partner, *past 6 mo*	58 (16%)	53 (18%)	5 (8%)
Other sleep disorders			
Obstructive sleep apnea	198 (55%)	173 (59%)	25 (38%)*
Central sleep apnea	29 (8%)	27 (9%)	2 (3%)
Restless legs syndrome	65 (18%)	53 (18%)	12 (18%)
Insomnia	101 (28%)	74 (25%)	25 (38%)
Periodic limb movement disorder	56 (16%)	45 (15%)	11 (17%)
Daytime somnolence	26 (7%)	22 (8%)	4 (6%)
Medication use			
Melatonin, *past or present use*	175 (49%)	148 (50%)	27 (41%)
Clonazepam, *past or present use*	133 (37%)	110 (38%)	22 (33%)
Antidepressant, *past or present use*	200 (55%)	151 (51%)	49 (74%)*
Antidepressant/RBD association	23 (6%)	17 (6%)	6 (9%)
Other, *past or present use*	51 (14%)	40 (14%)	11 (17%)
Sleep questionnaires			
SCOPA sleep (participant), *score*	11.8 ± 6.8	11.9 ± 6.41	11.3 ± 6.2
Day, *sub‐score*	3.4 ± 3.0	3.5 ± 3.03	3.0 ± 3.0
Night, *sub‐scor*e	8.4 ± 4.7	8.4 ± 4.67	8.3 ± 4.9
SCOPA sleep (co‐participant), *score*	12.5 ± 7.2	13.0 ± 7.3	10.4 ± 6.3*
Day, *sub‐score*	4.0 ± 3.6	4.2 ± 3.7	2.6 ± 2.6*
Night, *sub‐score*	8.6 ± 5.3	8.8 ± 5.4	7.7 ± 4.6
Epworth Sleepiness Scale, *score*	6.5 ± 4.7	6.5 ± 4.7	6.4 ± 4.9

Data are presented as mean ± standard deviation, or raw frequency count with percent of the total number of available responses in parentheses. Other psychotropic/neurologic/sleep‐related medications use include zolpidem, trazodone, cannabidiol, lorazepam, prazosin, ropinirole, donepezil, pimavanserin, diphenhydramine, and gabapentin.

RBDSS, RBD Severity Scale; SCOPA, Scales for Outcomes in Parkinson's Disease.

* *p* < 0.05 female versus male.

**Table 3:** RBD characteristics and sleep outcomes based on antidepressant use.

	Any use	No use
	*n* = 200	*n* = 154
Age, *years*	63.4 ± 10.5	67.0 ± 9.1
Sex, *male*	149 (75%)	140 (91%)*
Hypertension	80 (40%)	57 (37%)
Hypercholesterolemia	73 (37%)	19 (12%)
RBD behavior and characteristics		
Earliest age of onset, *years*	47.7 ± 17.6	55.8 ± 17.4*
Movement/talking	197 (99%)	149 (97%)
Movement/talking w/ dreams, *always*	114 (57%)	89 (58%)
RBD behavior w/o medication, *daily*	72 (36%)	50 (33%)
RBD behavior w/ medication, *daily*	45 (22%)	32 (21%)
RBD behavior: injured self, *ever*	113 (57%)	86 (56%)
RBD behavior: injured self, *6 m*o	54 (27%)	29 (19%)*
RBD behavior: injured bed partner, *ever*	84 (42%)	58 (38%)
RBD behavior: injured bed partner, *6 m*o	34 (17%)	23 (15%)
Medication use		
Melatonin, *past or present use*	77 (39%)	53 (34%)
Clonazepam, *past or present use*	91 (46%)	82 (53%)
Other, *past or present use*	46 (23%)	3 (2%)*
Other sleep disorders		
Obstructive sleep apnea	116 (58%)	74 (48%)
Restless legs syndrome	50 (25%)	14 (9%)*
Insomnia	67 (34%)	31 (20%)*
Periodic limb movement disorder	37 (19%)	18 (12%)
Sleep questionnaires		
SCOPA Sleep (participant), *score*	13.2 ± 6.7	9.9 ± 5.5*
SCOPA Sleep (co‐participant), *score*	13.8 ± 7.6	10.7 ± 6.3*
Epworth Sleepiness Scale, *score*	7.11 ± 5.0	5.6 ± 4.1*

Data are presented as mean ± standard deviation, or raw frequency count with percent of the total number of available responses in parentheses. Data missing on *n* = 6 participants. Other psychotropic/neurologic/sleep‐related medications use include zolpidem, trazodone, cannabidiol, lorazepam, prazosin, ropinirole, donepezil, pimavanserin, diphenhydramine, and gabapentin.

SCOPA, Scales for Outcomes in Parkinson's Disease.

* *p* < 0.05 versus any use.

**Table 4:** RBD characteristics and sleep outcomes in isolated RBD.

	Isolated RBD	RBD+
	*n* = 56	*n* = 305
Age, *years*	57.3 ± 13.4	66.3 ± 8.6*
Sex, *male*	37 (66%)	258 (85%)*
Hypertension	15 (27%)	125 (41%)*
Hypercholesterolemia	17 (30%)	116 (38%)
RBD behavior and characteristics		
Earliest age of onset, *years*	42.9 ± 20.9	52.5 ± 17.5*
Movement/talking	55 (98%)	297 (97%)
Movement/talking w/ dreams, *always*	33 (59%)	170 (56%)
RBD behavior w/o medication, *daily*	9 (16%)	115 (38%)*
RBD behavior w/ medication, *daily*	3 (5%)	77 (25%)*
RBD behavior: injured self, *ever*	23 (41%)	179 (59%)*
RBD behavior: injured self, *6 m*o	8 (14%)	77 (25%)
RBD behavior: injured bed partner, *ever*	18 (32%)	125 (41%)
RBD behavior: injured bed partner, *6 m*o	8 (14%)	50 (16%)
Medication use		
Melatonin, *past or present use*	19 (34%)	114 (37%)
Clonazepam, *past or present use*	25 (45%)	150 (49%)
Antidepressant, *past or present use*	23 (41%)	125 (41%)
Antidepressant/RBD association	7 (13%)	16 (5%)
Other, *past or present use*	13 (23%)	38 (12%)
Other sleep disorders		
Obstructive sleep apnea	35 (62%)	163 (53%)
Restless legs syndrome	6 (11%)	59 (19%)
Insomnia	19 (34%)	80 (26%)
Periodic limb movement disorder	7 (13%)	49 (16%)
Sleep questionnaires		
SCOPA Sleep (participant), *score*	12.2 ± 6.2	11.7 ± 6.4
SCOPA Sleep (co‐participant), *score*	11.0 ± 6.8	12.7 ± 7.2
Epworth Sleepiness Scale, *score*	6.9 ± 5.3	6.4 ± 4.6

Data are presented as mean ± standard deviation, or raw frequency count with percent of the total number of available responses in parentheses. See text for definition of isolated RBD. Other psychotropic/neurologic/sleep‐related medications use include zolpidem, trazodone, cannabidiol, lorazepam, prazosin, ropinirole, donepezil, pimavanserin, diphenhydramine, and gabapentin.

SCOPA, Scales for Outcomes in Parkinson's Disease.

* *p* < 0.05 versus isolated RBD.

**Table 5:** General, cardio/cerebrovascular, neurological, and mental health history.

	Whole Cohort	Male	Female
	*n* = 361	*n* = 295	*n* = 66
General health			
Hypercholesterolemia	133 (37%)	115 (39%)	18 (27%)*
Arthritis	116 (32%)	83 (28%)	33 (50%)*
Thyroid disease	51 (14%)	35 (12%)	16 (24%)*
Type II diabetes	42 (12%)	35 (12%)	7 (11%)
Vitamin B12 deficiency	30 (8%)	22 (8%)	8 (12%)
Seizures	10 (3%)	7 (2%)	3 (5%)
Cardiovascular and cerebrovascular			
Hypertension	140 (39%)	126 (43%)	14 (21%)*
Atrial fibrillation	38 (11%)	33 (11%)	5 (8%)
Myocardial infarction	20 (6%)	18 (6%)	2 (3%)
Stroke	10 (3%)	9 (3%)	1 (2%)
Other cardiovascular disease	42 (12%)	33 (11%)	9 (13%)
Autonomic function			
SCOPA‐AUT, *score*	13.5 ± 7.8	13.5 ± 7.5	13.3 ± 9.0
Urinary incontinence	60 (17%)	49 (17%)	11 (17%)
Bowel incontinence	13 (4%)	9 (3%)	4 (6%)
Traumatic brain injury			
Traumatic brain injury, *ever*	97 (27%)	86 (29%)	11 (17%)
With LOC <5 min	56 (16%)	49 (17%)	7 (11%)
With LOC >5 min	26 (7%)	22 (8%)	4 (6%)
Without LOC	40 (11%)	35 (12%)	5 (5%)
Recency, *years*	31 ± 18	31 ± 16	28 ± 15
Mental health			
BAI, score	8.1 ± 8.8	7.6 ± 8.4	10.3 ± 10.3
Anxiety, *self‐report*	142 (39%)	103 (35%)	39 (59%)*
PHQ‐9, *score*	5.2 ± 5.4	5.1 ± 5.3	6.0 ± 5.5
Depression, *<2 years self‐report*	112 (31%)	91 (31%)	21 (32%)
PCL‐5, *score*	12.3 ± 15.6	12.2 ± 15.5	12.7 ± 15.6
PTSD, *self‐report*	48 (13%)	40 (14%)	8 (12%)
Obsessive–compulsive disorder	21 (6%)	18 (6%)	3 (5%)
Developmental disorder	33 (9%)	25 (8%)	8 (12%)
Neuropsychiatric inventory			
Delusions	17 (4%)	15 (5%)	2 (3%)
Hallucinations	20 (6%)	18 (6%)	2 (3%)
Anxiety	80 (22%)	66 (22%)	14 (21%)
Apathy/indifference	72 (20%)	68 (23%)	4 (6%)*
Tobacco and alcohol use			
Tobacco, *within the past 30 days*	27 (7%)	24 (8%)	3 (5%)
Tobacco, *total years smoked*	63.6 ± 34.6	63.9 ± 35.1	62.2 ± 36.6
Alcohol, *within the past 90 days*	272 (75%)	224 (76%)	48 (73%)
Alcohol, *≥3 drinks per week*	68 (19%)	62 (21%)	6 (9%)

Data are presented as mean ± standard deviation, or raw frequency count with percent of the total number of available responses in parentheses. Other cardiovascular disease includes, arrhythmias/bundle branch block (*n* = 8), congenital heart defects (*n* = 4).

BAI, Beck Anxiety Inventory; BP, blood pressure; LOC, loss of consciousness; PCL‐5, post‐traumatic stress disorder checklist for DSM‐V; PHQ‐9, Patient Health Questionnaire‐9; SCOPA‐AUT, scales for Outcomes in Parkinson's Disease Autonomic Function.

* *p* < 0.05 female versus male.

**Table 6:** Cognition, motor, autonomic, and sensory function.

	Whole cohort	Abnormal	Male	Female
	*n* = 361		*n* = 295	*n* = 66
Cognition				
Montreal cognitive assessment	26.2 ± 3.0	116 (38%)	26.0 ± 5.3	27.1 ± 4.5*
Craft story				
Immediate verbatim	13.1 ± 5.2	99 (27%)	13.0 ± 5.1	13.3 ± 6.0
Delay verbatim	15.3 ± 6.6	93 (26%)	15.1 ± 6.7	16.3 ± 6.9
Benson				
Immediate	15.5 ± 1.4	50 (14%)	15.5 ± 2.1	15.6 ± 2.4
Delay	11.2 ± 3.2	56 (15%)	11.2 ± 3.4	10.9 ± 4.1
Number span				
Total forward	8.3 ± 2.4	23 (6%)	8.3 ± 2.4	8.4 ± 2.3
Total backward	6.8 ± 2.3	44 (12%)	6.7 ± 2.3	7.1 ± 1.9
Trails A*, seconds*	41.5 ± 73.6	99 (27%)	43.1 ± 80.9	34.3 ± 17.0
Trails B*, seconds*	103.5 ± 119.1	81 (22%)	106.0 ± 128.6	92.3 ± 58.7
Multilingual naming test	30.1 ± 2.1	47 (13%)	30.17 ± 2.6	29.6 ± 2.6*
Phonemic/categorical fluency				
F words	13.8 ± 5.1	46 (13%)	13.5 ± 5.2	15.1 ± 5.1*
L words	12.8 ± 5.2	61 (17%)	12.5 ± 5.2	14.1 ± 5.1*
Animals	20.3 ± 5.5	55 (15%)	20.1 ± 5.5	21.2 ± 5.7
Vegetables	14.0 ± 4.2	77 (21%)	13.5 ± 4.1	15.9 ± 3.9*
Speeded attention task				
Raw word	105.9 ± 127.6	–	106.3 ± 130.3	104.0 ± 113.7
Raw color	63.3 ± 14.9	–	62.8 ± 18.7	65.8 ± 17.7
Raw word + color	35.9 ± 13.0	–	35.4 ± 14.3	38.1 ± 15.1
Noise pareidolia task				
Yes face	6.93 ± 0.77	–	6.9 ± 0.9	6.9 ± 0.3
No noise	12.59 ± 3.82	–	12.4 ± 1.9	13.5 ± 8.0
Yes face + No noise	19.51 ± 3.81	–	19.3 ± 2.1	20.4 ± 8.1
Motor				
MDS‐UPDRS part 3, *score*	2.07 ± 3.46	72 (24%)	2.15 ± 3.5	1.73 ± 3.29
Purdue pegboard, *dominant hand*	10.88 ± 2.42	87 (29%)	10.67 ± 2.34	11.77 ± 2.56*
Alternate tap test, *dominant hand*	174 ± 40	145 (48%)	175 ± 41	172 ± 34
Timed up and go, *seconds*	9.01 ± 3.23	24 (8%)	8.89 ± 3.24	9.53 ± 4.06
Autonomic				
Supine BP, *mm Hg*	138 ± 19/80 ± 10	–	139 ± 20/81 ± 11	133 ± 22*/78 ± 11
Supine heart rate, *bpm*	64 ± 11	–	64 ± 12	66 ± 10
3 min standing BP, *mm Hg*	128 ± 18/80 ± 11	–	130 ± 28/81 ± 18	122 ± 37*/78 ± 23
3 min standing heart rate, *bpm*	73 ± 13	–	73 ± 18	76 ± 23*
Orthostatic hypotension (20–30/10–15)	51 (14%)	–	43 (15%)	9 (14%)
Orthostatic hypotension (30/15)	48 (13%)	–	34 (12%)	14 (21%)
Sensory				
Farnsworth‐Munsell color vision test	143.7 ± 97.6	226 (74%)	149.3 ± 101.0	119.2 ± 89.0*
Color blindness	22 (6%)	–	20 (7%)	2 (3%)
Brief smell identification test	7.2 ± 3.0	176 (57%)	7.0 ± 3.1	8.2 ± 2.7*
Olfactory symptoms	79 (22%)	–	63 (21%)	87 (24%)

Data are presented as mean ± standard deviation, or raw frequency count with percent of the total number of available responses in parentheses. Criteria for abnormality: Montreal Cognitive Assessment, Z‐score at or below 1.5 SD based on total score ≤ 25; MDS‐UPDRS part 3, based on total score > 4; Purdue Pegboard, based on total score < 9; Alternate Tap Test, based on total score < 165; Timed Up and Go, based on total time ≥ 13.5 s; Farnsworth‐Munsell Color Vision Test, based on total score > 100; Brief Smell Identification Test, based on total score ≤ 8.

BP, blood pressure; MDS‐UPDRS, Movement Disorder Society Unified Parkinson's Disease Rating Scale.

* *p* < 0.05 versus male.

## Results

### Demographics

The participant cohort (*n* = 361; Table 1) was predominately male (81.7%), middle/old‐aged (64.9 ± 10.1 years), white (90.3%), right‐handed (84.8%), married (77.0%), with >15 years of education (69.2%). Among statistically significant demographic differences between men and women, a greater proportion of men were married/living as a domestic partnership (80% vs. 63.6%, *p* = 0.004), while a greater proportion of women were divorced/separated (16.7% vs. 4.7%, *p* < 0.001) or were widowed (6.1% vs. 1.7%, *p* = 0.04).

### RBD characteristics and sleep

The overall cohort reported mean RBD symptom onset at age 51.1 ± 16.3 (Table 2). There were nine participants reporting an age of onset ≤16 years, possibly reflecting Parasomnia Overlap Disorder that progressed to RBD over time^45^; for the purpose of determining the group average, these were considered as 16 years of age. The pre‐adjusted group average was not significantly different at 50.6 ± 15.2 years of age. RBD behavior was commonly associated with prior injury to themselves (56.0%) and their bed partners (39.6%). Medication usage of interest included melatonin (48.5%) and clonazepam (36.8%). Antidepressant usage history was reported by 55.4%, of whom only 11.5% indicated antidepressant medication worsened RBD symptoms. Other current medications are listed in Table 2. Roughly half (54.8%) of the overall population had a history of obstructive sleep apnea (8.0% with central sleep apnea). There was a high frequency of insomnia (27.4%), restless legs syndrome (18.0%), and periodic limb movement disorder (15.5%). Participant SCOPA Sleep (total) scores averaged 11.8 ± 6.4, with bed partners/co‐participants reporting scores of 12.5 ± 7.2 for the participant, suggesting mild impairment on average. The mean Epworth Sleepiness Scale score was 6.5 ± 4.7, which is in the normal range.

With regard to sex differences, RBD symptom onset occurred ~3 years earlier in women than men, a difference that was not statistically significant (48.9 ± 21.0 vs. 51.6 ± 18.1 years of age; *p* = 0.244). Women reported fewer RBD‐related injuries, including to their bed partner (16.7% vs. 44.7%, *p* = 0.038). Despite the greater proportion of women reporting a history of antidepressant usage (74.2% vs. 51.2%; *p <* 0.001), women less frequently associated RBD behavior with medication use (24.7% vs. 10.6%; *p* = 0.027). None of these RBD‐related differences in women were associated with history of antidepressant usage. With respect to other sleep disorders, women reported higher rates of insomnia (37.9% vs. 25.1%, *p* = 0.038), and lower rates of obstructive sleep apnea (37.9% vs. 58.6%; *p* = 0.01), of which only the latter was statistically significant after adjustment for antidepressant usage. The only difference in self‐reported sleep scores was seen in the co‐participant's report of the SCOPA‐Sleep total score (10.4 ± 6.3 vs. 12.9 ± 7.3, *p* = 0.005) and daytime sub‐score (2.6 ± 2.6 vs. 4.2 ± 3.7, *p* = 0.005).

Antidepressant usage within the overall population was examined based on participants reporting any history of antidepressant usage (previous or current; no clinically relevant differences were noted when comparing only previous or only current usage) compared to participants with no history of antidepressant usage (Table 3). Despite similar age of enrollment, RBD symptom onset occurred 8.1 years earlier in participants with a history of antidepressant usage (47.7 ± 17.5 vs. 55.8 ± 17.4 years of age; *p <* 0.001). Restless legs syndrome (25.0% vs. 9.1%; *p* < 0.001), and insomnia (33.5% vs. 20.1%; *p* = 0.004) were more frequent in participants with antidepressant usage. Medications reported to worsen RBD symptoms primarily included antidepressants including selective serotonin reuptake inhibitors (SSRI; fluoxetine, paroxetine, sertraline, escitalopram), selective norepinephrine reuptake inhibitors (SNRI; venlafaxine, duloxetine), norepinephrine dopamine reuptake inhibitors (NDRI; bupropion), tri/tetracyclic antidepressants (TCA; amitriptyline, nortriptyline, mirtazapine, amitriptyline), and monoamine oxidase inhibitors (MAOI; phenelzine), anxiolytics (buspirone), dopamine agonists (pramipexole), and to a lesser extent, sedative‐hypnotics, and antihistamines (zolpidem, diphenhydramine, trazodone).

RBD and sleep‐related outcomes were compared between participants determined to have isolated RBD (i.e., those with no other signs of neurodegeneration) and those with possible neurodegenerative signs (i.e., RBD+; Table 4). Isolated RBD participants were younger (57.3 ± 13.5 vs. 66.3 ± 8.6, *p* < 0.001), reported an earlier age of RBD symptom onset (42.9 ± 20.9 vs. 52.5 ± 17.5, *p* < 0.001), and were more likely to be female (*p* < 0.001). Isolated RBD participants less frequently associated RBD behavior with medication usage (5.4% vs. 25.2%, *p* < 0.001), and reported lower rates of RBD behavior being associated with self‐injury (41.1% vs. 58.7%, *p* = 0.011). Differences may be explained by the higher proportion of women (and greater rate of antidepressant use) in the isolated RBD group, as there was no difference in the duration between symptom onset/diagnosis and NAPS referral with respect to isolated RBD versus RBD+, men versus women.

### Health history

The most commonly reported health problems in the cohort (Table 5) were hypercholesterolemia (36.8%), arthritis (32.1%; primarily osteoarthritis), hypertension (38.8%), thyroid disease (14.1%), type II diabetes (11.6%), and atrial fibrillation (10.5%). Women reported ~40% lower rate of hypercholesterolemia (27.3% vs. 39.0%; *p* = 0.05) and hypertension (21.2% vs. 42.7%; *p* = 0.001), and ~50% higher rate of arthritis (50.0% vs. 28.1%; *p* < 0.001) and thyroid disease (24.2% vs. 11.9%; *p* = 0.009) than men.

Autonomic function, as assessed by SCOPA‐AUT, averaged 13.5 ± 7.8 in the overall cohort, not significantly different between men and women. Urinary and bowel incontinence, as well as orthostatic hypotension were not different between men and women. Men reported a nearly two‐fold higher rate of sexual dysfunction compared to women (68% vs. 37%).

Approximately 27% of the cohort reported a history of traumatic brain injury (TBI) occurring on average 31 years ago, which was more common in men (29.2% vs. 16.7%; albeit non‐significant *p* = *0.056*). Similarly, TBI‐related metrics (i.e., with or without loss of consciousness) were also potentially higher in men than women.

The most common mental health problems were anxiety (39.3%) and depression (recent, <2 years ago, 31.0%; or inactive, >2 years ago, 29.5%). Individuals self‐reporting anxiety and depression averaged 14.4 ± 11.4 and 9.1 ± 6.1 for BAI and PHQ‐9 scores, respectively. However, self‐reported BAI and PHQ‐9 scores in the overall group averaged 8.1 ± 8.8 and 5.2 ± 5.4, respectively, which are scores below clinical thresholds for anxiety or depression (BAI scores <16 indicate mild to no anxiety; PHQ‐9 scores <10 indicate mild to no depression). Considering previously reported associations between PTSD and RBD,^46^ the overall rate of PTSD was 24.1% in this cohort, with no difference between men and women.

The Neuropsychiatric Inventory assessed domains related to delusions, hallucinations, anxiety, and apathy/indifference. This cohort had low rates of current delusions or hallucinations (~4%–5%), whereas anxiety was reported at 22.2% and apathy/indifference at 19.7%. Although men had higher rates in the “apathy/indifference” (22.7% vs. 6.1%; *p* < 0.001) domain, no sex‐related differences were found in the other three features.

### Neurological test battery results

**Figure 1:**
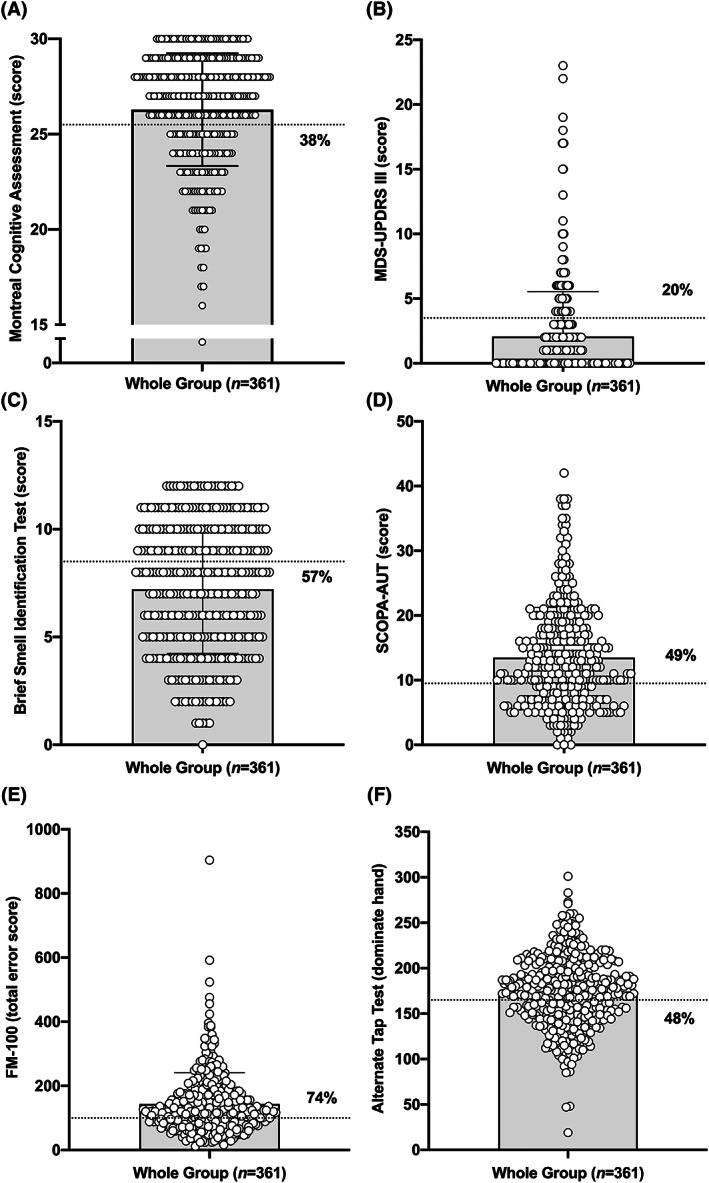
Distribution of scores in primary domains of function. The distribution of individual scores (open circles) with the mean value indicated via the shaded bar and the threshold for abnormality (dotted line) with the percentage of the total cohort falling above/below this threshold and meeting criteria for abnormal function for (A) the Montreal Cognitive Assessment (% abnormal based on Z‐score of 1.5 SD or below after normalization), (B) the MDS‐UPDRS part III (% abnormal based on total score > 4), (C) the Brief Smell Identification Test (% abnormal based on total score ≤ 8), (D) the SCOPA‐AUT (% abnormal based on total score > 13), (E) Farnsworth‐Munsell 100 color vision test (% abnormal based on total score > 100), (F) the Alternate Tap Test for participants dominant hand (% abnormal based on total score < 165).

The cognitive battery revealed relatively normal mean scores in the overall cohort, with women having better scores in the MoCA (27.1 ± 4.5 vs. men 26.0 ± 5.3; *p* = 0.011), worse scores on the MINT (29.6 ± 2.6 vs. 30.2 ± 2.6; *p* = 0.047), and improved phonemic/categorical verbal fluency (Table 6; Fig. 1). However, this result is inclusive of participants categorized as having isolated RBD, who, by definition, are not cognitively impaired and did not have abnormal psychometric test performance. Indeed, 38.3% of participants (*n* = 116) with RBD+ had MoCA scores ≤25 (threshold for defining abnormal scores^47^), and 27.6% of these individuals (*n* = 32) met criteria for mild cognitive impairment.^48^

Mean motor test and examination scores were in the normal range (Table 6; Fig. 1); however, among RBD+ participants, ~8% were abnormal on the TUG (defined as ≥13.5 s), ~30% were abnormal on the Purdue pegboard test (defined as <9), ~50% were abnormal on the alternate tap test (defined as <165) and ~25% were abnormal on the MDS‐UPDRS part 3 assessment (defined by scores >4).

A relatively large proportion of participants had autonomic impairment as assessed by orthostatic tolerance testing. Orthostatic hypotension was present in 27.7% of the cohort (defined by a systolic and/or diastolic blood pressure drop of >20/10 mm Hg, respectively, after 3 min of standing), with 13.3% having values in the severe range (i.e., systolic and/or diastolic drop >30/15 mm Hg); there were no differences between men and women.

Average FM‐100 scores in non‐color‐blind participants were 143.6 ± 97.6, indicating 74% of the cohort was abnormal, with women showing slightly better mean color vision (119 ± 89 vs. 149 ± 101; *p* = 0.024) (Table 6; Fig. 1). Consistent with known sex differences, there was a lower frequency of color blindness in women compared to men (3.0% vs. 6.8%; albeit non‐significant *p* = 0.26). Olfactory impairment was self‐reported in 21.9% of the overall population, however, 57% showed abnormal BSIT scores. Similarly, women had better olfaction than men on the BSIT (8.2 ± 2.7 vs. 7.0 ± 3.1; *p* = 0.004). Among RBD+ participants, ~50% were abnormal in both color vision and olfactory function.

### Family history

**Figure 2:**
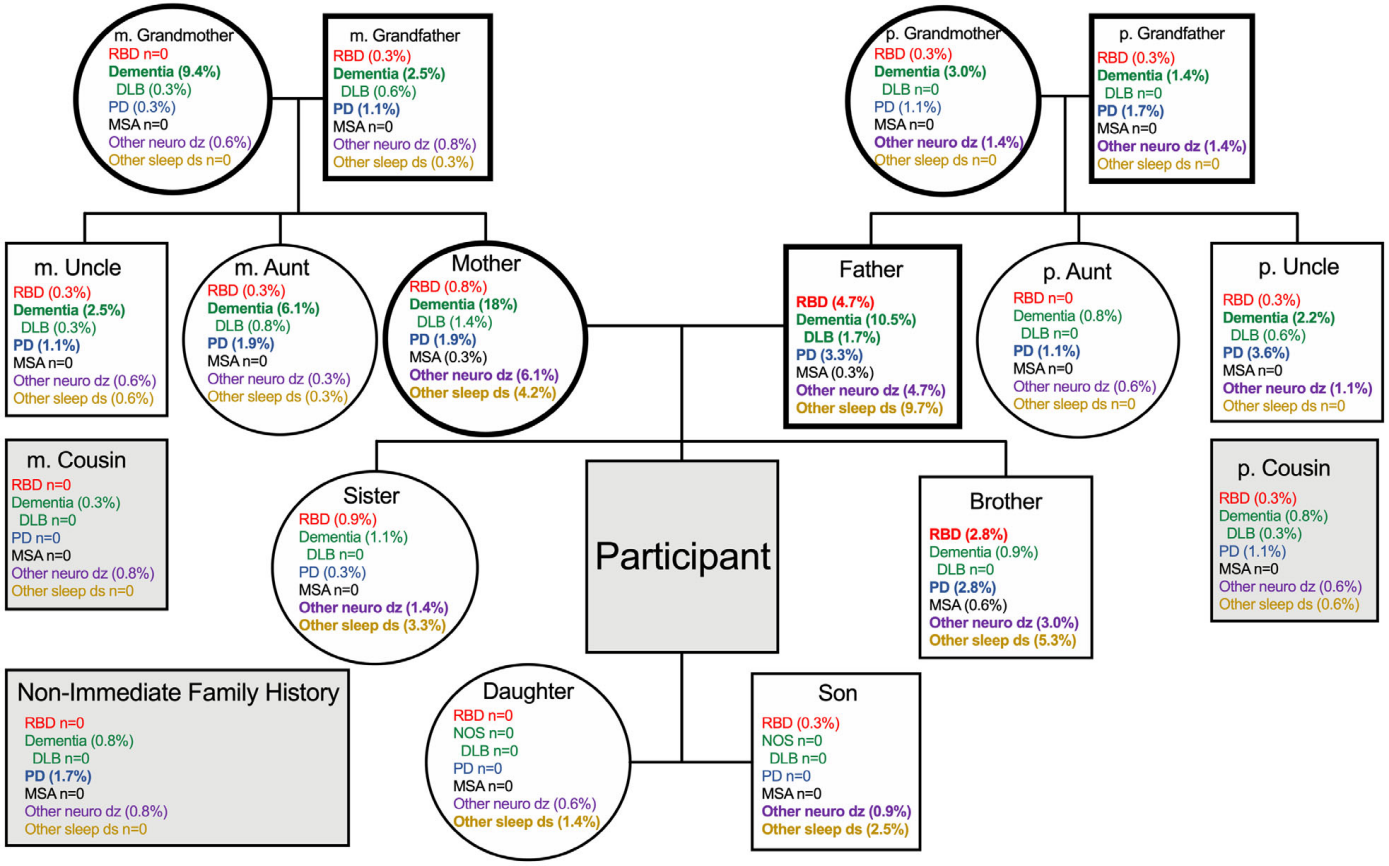
Genealogy of known RBD and related neurologic and sleep disorders. Participant (shaded center box) with offspring, sibling, and maternal (m.) and paternal (p.) family members. Non‐shaded circles indicate female sex and non‐shaded squares indicate male sex (shaded squares for participant, cousins and non‐immediate family imply male or female). Rates for RBD, Dementia (including: Alzheimer's Disease (AD), Dementia with Lewy Bodies (DLB), and Dementia NOS [no other symptoms]), Parkinson's Disease (PD), Multiple Systems Atrophy (MSA), other neurological diseases (dz), and other sleep disorders (ds) are listed within each family member. Within the Dementia category, the rate of DLB is indented and indicated. Rates above 1% are bolded for clearer visualization. Note: Raw frequency counts are not embedded due to (1) space constraints, and (2) limitations inherent to these data. All percentages reflect a total sample size of *n* = 361. However, this is only strictly applicable to participants mother/father, and m./p. grandparents, with the greater confidence in these categories denoted by thicker borders. Not all participants have siblings, offspring, m./p. aunts/uncles, cousins, or other non‐immediate family members. Further, participant “unknown” responses were not recorded.

Participants in this study provided extensive family history information (Fig. 2), however, these data rely on their individual knowledge and recall for these specific conditions (responses for “unknown” were not recorded). Familial history for probable RBD was predominant in males, with 2.6% of brothers and 4.7% of fathers reportedly having probable RBD, compared to a rate of 0.8% for sisters and mothers each. The rate of probable RBD in grandparents was consistent across paternal and maternal sides (0.3%). PD and DLB showed a male association (PD 3.2%, DLB 1.8% in fathers vs. PD 2.1% and DLB 1.3% in mothers). No evident maternal or paternal association was seen for MSA or other neurological disorders. The rate of the broad categorization of other sleep disorders was 9.5% in fathers and 4.5% in mothers. Alzheimer's disease and dementia NOS showed a stronger maternal association (16.9% in mothers and 9% in fathers), which was consistent in the previous generation (9.7% in maternal grandmothers, compared to 1%–3% in maternal grandfathers, paternal grandmothers, and paternal grandfathers).

## Discussion

We report characteristics of the initial NAPS Consortium cohort as measured through a standardized, comprehensive clinical assessment. Consistent with previous literature, we found a male preponderance for RBD (~80%),^49^, ^50^, ^51^, ^52^ and we confirmed prior findings that suggested antidepressant medications are associated with RBD presenting earlier in life and in women. We identified a high rate of subtle neurological dysfunction in RBD, with 84% of our cohort having an abnormality in at least one neurological domain. We also describe a comprehensive neurological family history in RBD, with RBD and PD family history higher in male relatives, and dementia family history higher in female relatives.

Even across 9 sites, there was a high male:female ratio in our RBD cohort, yet a striking lack of differences between men and women except for younger age of onset and higher antidepressant usage in women. Previous work has described an earlier age of RBD onset in women that can be attributed to previous or current antidepressant usage.^51^, ^53^ Indeed, in the present study the average age of men and women did not differ, and potential associations between metrics related to RBD severity and sex were associated with a higher rate of antidepressant usage among women. Of note, there was no sex difference in the duration of time between self‐reported RBD symptom onset and enrollment in NAPS, which could have resulted in an earlier age of onset in women if they were being referred more expeditiously than men. Other sex‐related differences were largely expected based upon known patterns of sex‐specific health conditions, for example, a higher frequency of OSA, hypertension, hypercholesterolemia, and history of TBI in men, compared to a higher rate of arthritis and thyroid disease in women. The higher rate of sexual dysfunction in men compared to women is relevant given sexual dysfunction is one of the earliest signs of autonomic impairment and neurodegeneration. However, sexual dysfunction is commonly poorly tracked in women and may not have been accurately assessed in these participants using the SCOPA‐AUT.

Antidepressants are known to cause changes in sleep architecture and polysomnography findings, predominantly during REM sleep (e.g., induce prominent eye movements, suppress REM sleep, and increase REM sleep without atonia, RSWA).^54^, ^55^, ^56^, ^57^ Although no studies have prospectively examined polysomnography findings before and after initiating antidepressant treatment, several studies have reported participants with RBD onset occurring shortly after initiating antidepressant treatment.^58^, ^59^, ^60^ It remains unknown if antidepressant use is unmasking the same neuropathologic process present in typical RBD, or if the pathophysiology differs. Longitudinal follow‐up studies, such as those proposed in the second stage of NAPS (i.e., NAPS2) will be necessary to address this controversial and important clinical question. We differentiated participants in the current study based on any previous history of antidepressant usage, given that it is currently unknown how long RBD/RSWA can persist after discontinuation of the presumed offending medication and whether or not long‐term usage is associated with a permanent/persistent upregulation of serotonergic and/or noradrenergic systems. In addition to RBD, insomnia, restless legs syndrome and periodic limb movements were also associated with a history of antidepressant usage.

“Soft” neurological abnormalities were very common in our cohort (i.e., RBD+ participants; *n* = 305 or 84%). We confirm that individuals with such findings are appropriate for inclusion in clinical trials since such features are common among RBD patients. We compared RBD+ participants with isolated RBD, who had no neurological abnormalities identified during a very detailed and comprehensive evaluation. Except for higher rate of restless legs syndrome and periodic limb movement disorders in RBD+, there were no other differences between the groups except older age in RBD+ and higher rate of antidepressant usage in the isolated RBD group. These data suggest that isolated RBD participants may be at an earlier stage of prodromal synucleinopathy rather than having a separate underlying etiology, although this will require longitudinal follow‐up for confirmation. Prospective comprehensive evaluations in both groups will provide insights on if, when, and to what phenotype participants with RBD +/− other neurodegenerative signs/symptoms will evolve. The NAPS Consortium is developing a clinical rating scale that encompasses the broad range of neurological dysfunction that is detectable during the prodromal phase of synucleinopathies. We anticipate that this Prodromal Synucleinopathy Rating Scale (PSRS) will serve as a clinical measure of synucleinopathy burden that can be used in neuroprotective clinical trials in RBD. Additionally, all willing current NAPS participants, and future enrollees, will undergo an expanded clinical and biomarker assessment as part of the NAPS Stage 2 (NAPS2) protocol (U19 AG071754) to develop additional biomarkers and achieve readiness for neuroprotective clinical trials.

The possible genetic underpinnings of RBD remain ill‐defined. Previous work reported a family history for dream enactment via the RBD1Q^61^ to be 13.8% in idiopathic RBD. In the NAPS Consortium cohort, a male preponderance was present in reported RBD family history, 4.7% of fathers versus 0.8% of mothers (Fig. 2). This is consistent with the sex distribution among the probands. Similarly, reported PD was more common among male relatives, while AD and dementia diagnoses were more common in women. While these differences may reflect “expected” sex effects on phenotypic manifestations of synucleinopathies, it remains possible that other unknown mechanisms are potentially contributing. Additional work is needed in better describing the genealogy of RBD, as several limitations to the present data exist. Namely, that participant “unknown” responses, as well as a detailed history of extended family (e.g., presence/absence of siblings, offspring, and aunts/uncles), were not recorded. As such, frequency counts may not accurately reflect true percentages. Data pertaining to participants mother/father and m./p. grandparents likely carry the greatest confidence with respect to frequency counts. All data collected reflected in Fig. 2 are intended to serve as an initial description of this important topic.

Limitations in this study include the cross‐sectional and descriptive nature inherent to these data and experimental design. Participants were recruited primarily from clinics offered on a consecutive basis and supplemented with community referrals through the www.naps‐rbd.org website. This introduced possible selection bias and may have contributed to the relatively demographically homogenous sample. Information pertaining to individuals who were not enrolled were not retained, and therefore, our understanding of how this primarily clinic‐based cohort might compare to a community‐based sample remains limited. As previously mentioned, NAPS2 intends to follow these participants longitudinally, with the addition of biofluid, neuroimaging, and other biomarkers. Additionally, data were collected across 10 different sites, and therefore, despite rigorous standardization, inter‐site variability may exist. Although this concern is tempered from our rigorous adjudication process, it remains possible that non‐detectable inter‐site variability between outcomes exists, and as such, could theoretically be a factor for any outcome. Antidepressant use history was obtained from participants' report and therefore subject to recall bias. However, our findings are consistent with existing literature on the effects of antidepressants on RBD/RSWA. Family history information is limited by relying completely upon participant recall; additional genetic investigations in the NAPS Consortium and other RBD cohorts will be invaluable in exploring genetic risks factors for synucleinopathies. Lastly, the present manuscript is intended to be a descriptive report of the NAPS Consortium baseline cohort, and as such, the inclusion of *p*‐values throughout the presentation of results should be interpreted knowing that they are not corrected for multiple comparisons, and that issues surrounding selection and confounding were not robustly modeled.

In summary, this multisite dataset represents a coordinated effort to enroll, characterize, and follow a cohort of hundreds of subjects with RBD across North America. Initial results from this current baseline characteristics study already reveal novel insights about sex differences, antidepressant use, heritability, and the breadth and severity of neurological impairment among individuals with idiopathic RBD. Future work is poised to answer critical questions about longitudinal progression of disease, pathophysiological features, biomarkers, and predictors of phenoconversion. Importantly, longitudinal data on this well‐characterized cohort will inform the design of future neuroprotective clinical treatment trials, especially those targeting the earliest phase of synucleinopathy.

## Author Contributions

Conception and design of the study. JEE, MML, ATK, RBP, AP, JFG, EKSL, LKF, JAF, DLB, DEH, AYA, MJH, CHS, JM, SRC, AV, EHD, MGM, DRS, JKLI, BFB, YSJ. Acquisition and analysis of data. JEE, MML, ATK, RBP, AP, JFG, EKSL, LKF, JAF, DLB, DEH, AYA, MJH, CHS, JM, SRC, AV, EHD, MGM, DRS, JKLI, BFB, YSJ. Drafting a significant portion of the manuscript or figures (i.e., a substantial contribution beyond copy editing and approval of the final draft, which is expected of all authors). JEE, MML, ATK, RMP, BFB, YSJ.

## Conflict of Interest

- Dr. Elliott has received support from the Department of Veteran Affairs, NIH (NHLBI, NIA, NCCIH), Oregon Medical Research Foundation, Portland VA Research Foundation, Eugene & Clarissa Evonuk Foundation in Environmental Physiology, and American Heart Association.
- Dr. Lim has received support from Department of Veteran Affairs, Department of Defense, NIH (NIMH, NHLBI, NIA, NCCIH, NINDS, NIGMS, NCATS), NSF, Center for Neuroscience & Regenerative Medicine (Henry Jackson Foundation), Oregon Medical Research Foundation, Collins Medical Trust, Brain & Behavior Foundation (NARSAD), American Sleep Medicine Foundation, Hartford Center of Gerontological Excellence, Pacific Northwest National Laboratory, and Portland VA Research Foundation.
- Allison Keil, Dr. Pelletier, Dr. Forsberg, Jennifer McLeland has no disclosures.
- Dr. Postuma has received support from the Fonds de Recherche du Quebec – Santé, the Canadian Institutes of Health Research, the Parkinson Society of Canada, the Weston‐Garfield Foundation, the Michael J. Fox Foundation, the Webster Foundation; and personal fees from Takeda, Roche/Prothena, Teva Neurosciences, Novartis Canada, Biogen, Boehringer Ingelheim, Ther‐ anexus, GE HealthCare, Jazz Pharmaceuticals, Abbvie, Jannsen, Otsuko, Phytopharmics, Inception Sciences, and Curasen.
- Dr. Gagnon has received support from the NIH, the Canadian Institutes of Health Research and the *Fonds de Recherche du Québec – Santé*.
- Dr. St. Louis has received support from NIH (NIA, NINDS, and NHLBI), the Michael J. Fox Foundation, Harmony, Inc., and Sunovion, Inc.
- Dr. Fields has received support from the NIH.
- Dr. Bliwise has received support from the NIH and has been a Consultant to CliniLabs, Eisai, Ferring, Huxley, Idorsia, and Merck.
- Dr. Huddleston has received support from NIH (NIA, NINDS, Department of Veteran Affairs, the American Parkinson's Disease Association Center for Advanced Research, the Emory Udall Parkinson's Disease Research Center, the Emory Lewy Body Dementia Association Research Center of Excellence, the Emory Alzheimer's Disease Research Center, the Michael J Fox Foundation, the Georgia Research Alliance, the Bumpus Family Foundation, and the McMahon Family).
- Dr. Avidan has received consultant fees from Avadel, Merck, Takeda, Eisai, Idorsia, and Harmony, and speaker honoraria from Merck, Eisai, Harmony, and Idorsia.
- Dr. Howell has received research support from the NIH.
- Dr. Schenck has received a one‐time speaker honorarium from Eisai, Inc.
- Dr. Criswell has received support from the NIH and consulting fees from Abbvie and Sio Gene Therapies.
- Dr. Videnovic has received research support from the NIH and the Michael J Fox Foundation; consultancy fees from Alexion Pharmaceuticals, Biogen, XW Pharma, Jazz.
- Dr. During has received support from Jazz Pharmaceuticals, Sanofi, Takeda, Rythm Inc., and the Feldman Foundation CA.
- Dr. Miglis has received support from Jazz Pharmaceuticals, Embr Wave, and Biohaven Pharmaceuticals; Consulting fees from 2nd MD, Infinite MD, and Guidepoint LLC; Payments for CME lectures from MED‐IQ; Royalties from Elsevier Inc.
- Dr. Shprecher has received support from the Arizona Alzheimer's Consortium, Abbvie, Acadia, Aptinyx, Axovant, Biogen, Eisai, Eli Lilly, Enterin, Neurocrine, Michael J Fox Foundation, NIH, Nuvelution, Theravance, and Teva; consultant fees from Amneal, Forensis, and Neurocrine; speaker honoraria from Acorda, Amneal, Neurocrine, Sunovion, Teva, and US World Meds/Supernus.
- Dr. Lee‐Iannotti has received support from NIH, Liva Nova, Respicardia, and the Arizona Alzheimer's Consortium. She serves on the Scientific Advisory Board for Jazz Pharmaceuticals, INSPIRE, and Avadel. She is a consultant and speaker for Jazz Pharmaceuticals.
- Dr. Boeve has served as an investigator for clinical trials sponsored by Alector and Biogen. He serves on the Scientific Advisory Board of the Tau Consortium. He receives support from NIH, the Mayo Clinic Dorothy, and Harry T. Mangurian Jr. Lewy Body Dementia Program, the Little Family Foundation, and the Ted Turner and Family Foundation.
- Dr. Ju has received support from the NIH and the Centene Corporation contract (P19‐00559) for the Washington University‐Centene ARCH Personalized Medicine Initiative; and compensation for consultant activities for Applied Cognition.

## APPENDIX A

**NAPS Consortium Personnel:**

**Co‐principal Investigators:** Yo‐El S. Ju and Bradley F. Boeve.

**Site Investigators (*alphabetical*):** Alon Y. Avidan, Donald L. Bliwise, Parichita Choudhury, Susan R. Criswell, Emmanuel H. During, Jonathan E. Elliott, Julie A. Fields, Leah K. Forsberg, Jean‐François Gagnon, Michael J. Howell, Daniel E. Huddleston, Joyce K. Lee‐Iannotti, Miranda M. Lim, Mitchell G. Miglis, Ronald B. Postuma, David R. Shprecher, Erik K. St. Louis, Aleksandar Videnovic.

**Study Coordinators and Research Associates:** Jennifer McLeland (lead), Sommer Amudson‐Huffmaster, Nellie Brushaber, Jae Woo Chung, Joshua De Kam, Adrian Ekelmans, Marissa Keane, Allison T. Keil, Ruth Kraft, Brittany R. Ligman, Colum MacKinnon, Daeva Miner‐Rose, Samantha Murphy, Amelie Pelletier, Katherine L. M. Powers, Matthew Stauder, Adreanne Rivera, Sarahmay Sanchez, Rebekah Summers, Luke Tiegan, Paul Timm, Kelsey A. Tucker.

**Data management:** Peter Tran.

**Consultants:** Douglas Galasko, Emmanuel Mignot, Carlos Schenck.

*The interpretations and conclusions expressed in this article are those of the authors and do not necessarily reflect the position or policy of the Department of Veterans Affairs, the National Institute of Health, or the United States government*.
